# Randomized Controlled Trial of the Effect of an Exercise Rehabilitation Program on Symptom Burden in Maintenance Hemodialysis: A Clinical Research Protocol

**DOI:** 10.1177/20543581241234724

**Published:** 2024-04-03

**Authors:** Emilie Ford, Krista Stewart, Eric Garcia, Monica Sharma, Reid Whitlock, Ruth Getachew, Krista Rossum, Todd A. Duhamel, Mauro Verrelli, James Zacharias, Paul Komenda, Navdeep Tangri, Claudio Rigatto, Jennifer M. MacRae, Clara Bohm

**Affiliations:** 1 Department of Internal Medicine, Max Rady College of Medicine, University of Manitoba, Winnipeg, MB, Canada; 2 Chronic Disease Innovation Centre, Seven Oaks General Hospital, Winnipeg, MB, Canada; 3 Manitoba Renal Program, Winnipeg, MB, Canada; 4 Faculty of Kinesiology and Recreation Management, University of Manitoba, Winnipeg, MB, Canada; 5 Institute of Cardiovascular Sciences, St. Boniface General Hospital Albrechtsen Research Centre, Winnipeg, MB, Canada; 6 Department of Cardiac Sciences, Cumming School of Medicine, University of Calgary, AB, Canada

**Keywords:** hemodialysis, end-stage kidney disease, exercise, symptom burden, health-related quality of life

## Abstract

**Background:**

People receiving hemodialysis experience high symptom burden that contributes to low functional status and poor health-related quality of life. Management of symptoms is a priority for individuals receiving hemodialysis but limited effective treatments exist. There is emerging evidence that exercise programming can improve several common dialysis-related symptoms.

**Objective:**

The primary aim of this study is to evaluate the effect of an exercise rehabilitation program on symptom burden in individuals receiving maintenance hemodialysis.

**Design:**

Multicenter, randomized controlled, 1:1 parallel, open label, prospective blinded end point trial.

**Setting:**

Three facility-based hemodialysis units in Winnipeg, Manitoba, Canada.

**Participants:**

Adults aged 18 years or older with end-stage kidney disease receiving facility-based maintenance hemodialysis for more than 3 months, with at least 1 dialysis-related symptom as indicated by the Dialysis Symptom Index (DSI) severity score >0 (n = 150).

**Intervention:**

Supervised 26-week exercise rehabilitation program and 60 minutes of cycling during hemodialysis thrice weekly. Exercise intensity and duration were supervised and individualized by the kinesiologist as per participant baseline physical function with gradual progression over the course of the intervention.

**Control:**

Usual hemodialysis care (no exercise program).

**Measurements:**

Our primary outcome is change in symptom burden at 12 weeks as measured by the DSI severity score. Secondary outcomes include change in modified DSI severity score (includes 10 symptoms most plausible to improve with exercise), change in DSI severity score at 26 and 52 weeks; time to recover post-hemodialysis; health-related quality of life measured using EuroQol (EQ)-5D-5L; physical activity behavior measured by self-report (Godin-Shepherd questionnaire) and triaxial accelerometry; exercise capacity (shuttle walk test); frailty (Fried); self-efficacy for exercise; and 1-year hospitalization and mortality.

**Methods:**

Change in primary outcome will be compared between groups by independent 2-tailed *t* test or Mann-Whitney U test depending on data distribution and using generalized linear mixed models, with study time point as a random effect and adjusted for baseline DSI score. Similarly, change in secondary outcomes will be compared between groups over time using appropriate parametric and nonparametric statistical tests depending on data type and distribution.

**Limitations:**

The COVID-19 pandemic restrictions on clinical research at our institution delayed completion of target recruitment and prevented collection of accelerometry and physical function outcome data for 15 months until restrictions were lifted.

**Conclusions:**

The application of an exercise rehabilitation program to improve symptom burden in individuals on hemodialysis may ameliorate common symptoms observed in individuals on hemodialysis and result in improved quality of life and reduced disability and morbidity over the long term. Importantly, this pragmatic study, with a standardized exercise intervention that is adaptable to baseline physical function, addresses an important gap in both clinical care of hemodialysis patients and our current knowledge.

## Abrege

**Contexte:**

Les personnes sous hémodialyse éprouvent un grand nombre de symptômes qui contribuent à un faible état fonctionnel et à une mauvaise qualité de vie liée à la santé. La prise en charge des symptômes est une priorité pour les personnes sous hémodialyse, mais les traitements efficaces sont limités. De nouvelles preuves montrent que l’adoption d’un programme d’exercice permettrait d’améliorer plusieurs symptômes courants liés à la dialyse.

**Objectifs:**

Le principal objectif de cette étude est d’évaluer l’effet d’un programme de rééducation par l’exercice sur le fardeau des symptômes chez les personnes recevant une hémodialyse d’entretien.

**Conception:**

Essai clinique prospectif randomisé-contrôlé, en aveugle, en parallèle 1:1 et ouvert, multicentrique.

**Cadre:**

Trois unités d’hémodialyse de Winnipeg, au Manitoba (Canada).

**Sujets:**

Des adultes atteints d’insuffisance rénale terminale qui reçoivent des traitements d’hémodialyse d’entretien en centre depuis plus de trois mois et qui présentent au moins un symptôme lié à la dialyse, tel qu’indiqué par un score de gravité de l’indice des symptômes de la dialyse (Dialysis Symptom Index) supérieur à zéro (n = 150).

**Intervention:**

Programme supervisé de rééducation par l’exercice d’une durée de 26 semaines et 60 minutes de vélo trois fois par semaine pendant l’hémodialyse. L’intensité et la durée de l’exercice ont été supervisées par un kinésiologue qui les a ensuite personnalisées en fonction de la forme physique initiale du participant en prévoyant une progression graduelle tout au long de l’intervention.

**Groupe témoin:**

Soins habituels d’hémodialyse (sans programme d’exercice).

**Mesures:**

Notre principal critère de jugement est un changement dans le fardeau lié aux symptômes après 12 semaines, tel que mesuré par le score de gravité de l’indice des symptômes de dialyse (ISD). Les critères d’évaluation secondaires comprennent un changement du score modifié de gravité de l’ISD (portant sur 10 symptômes les plus plausibles de s’améliorer avec l’exercice), la modification du score de gravité de l’ISD après 26 et 52 semaines, le temps de récupération après l’hémodialyse, la qualité de vie liée à la santé mesurée par le questionnaire EQ5D-5L, le comportement lié à l’activité physique mesuré par autoévaluation (questionnaire Godin-Shepherd) et par accéléromètre triaxial, la capacité d’effort (test de marche navette), la fragilité (Fried), le sentiment d’efficacité autodéclaré face à l’exercice, ainsi que les hospitalisations et la mortalité à un an.

**Méthodologie:**

Les changements pour le principal critère de jugement seront comparés entre les groupes par un test t bilatéral indépendant ou un test U de Mann-Whitney en fonction de la distribution des données, ainsi qu’à l’aide de modèles linéaires mixtes généralisés avec un point temporel de l’étude comme effet aléatoire et corrigé en fonction du score ISD initial. Les changements dans les résultats secondaires seront comparés entre les groupes au fil du temps à l’aide des tests statistiques paramétriques et non paramétriques appropriés selon le type de données et la distribution.

**Limites:**

Les restrictions liées à la pandémie de COVID-19 dans notre établissement ont retardé le recrutement des cibles et empêché pendant 15 mois la collecte de données sur les résultats mesurés par l’accéléromètre et les mesures de la fonction physique, soit jusqu’à ce que les restrictions soient levées.

**Conclusion:**

L’adoption d’un programme de rééducation par l’exercice visant à réduire le fardeau lié aux symptômes chez les personnes sous hémodialyse peut améliorer les symptômes courants observés dans cette population et se traduire par une amélioration de la qualité de vie et une réduction de l’invalidité et de la morbidité à long terme. Il convient de noter que cet essai pragmatique, avec son intervention d’exercice standardisée adaptable à la condition physique initiale de la personne, comble une lacune importante dans les soins cliniques des patients sous hémodialyse et dans nos connaissances actuelles.

## Introduction

Individuals with end-stage kidney disease (ESKD) require kidney replacement therapy to sustain life. Globally, the majority of individuals who enter this stage start facility-based hemodialysis.^1^ Individuals receiving hemodialysis have low self-reported functional status, and poor health-related quality of life (HRQOL) when compared with general population controls.^2^ Functional status is an important component of HRQOL and refers to the ability to perform activities required for daily life.

Symptom burden, the combined impact of the number and severity of symptoms experienced by an individual, is high in people receiving maintenance hemodialysis and contributes to low functional status and poor HRQOL.^2^ Cross-sectional studies report that 30% to 80% of people receiving dialysis have at least 1 symptom that is related to ESKD or dialysis treatment, with a mean of 6 to 20 symptoms endorsed per individual.^3-7^ In a systematic review of symptom burden in chronic kidney disease, fatigue was identified as the most widespread symptom, with a weighted mean prevalence of 81%.^4^ Frequent symptoms (>50% prevalence) also include lack of sleep, pain, muscle cramping, pruritus (itch), and decreased appetite, among others.^2,4^

Unfortunately, hemodialysis treatment itself does little to improve symptom burden and may worsen symptoms. In a study of approximately 700 people followed for 1 year after starting hemodialysis, symptoms worsened in 23%, did not change in 56%, and improved in only 19% of patients over the first year.^8^ Furthermore, health care providers underrecognize symptoms and underestimate symptom severity in people receiving hemodialysis in the majority of cases.^4^ Even when recognized, limited effective treatments exist for most symptoms. For those few symptoms for which there are effective pharmacological therapies, people receiving hemodialysis may be hesitant to take additional medications due to preexisting medication burden and concerns regarding side effects.^9-11^

Most importantly, the identification of effective treatments for hemodialysis-related symptoms has been identified as an important knowledge gap and research priority by multiple stakeholders, including people receiving hemodialysis and their caregivers.^12^ In further prioritizing studies, exercise has been identified as a candidate intervention to explore as treatment for several common hemodialysis-related symptoms, including fatigue, sleep disorders, and cramping.^13^

Improvement of symptom burden with exercise is biologically plausible and supported by evidence in the literature. Increased prevalence of symptoms is correlated with low physical activity in individuals on dialysis.^14,15^ Even in low-functioning hemodialysis patients, exercise programming is safe and improves outcomes closely associated with symptom burden, such as functional status and HRQOL.^16,17^ A recent systematic review and meta-analysis of 15 randomized controlled trials (RCTs) investigated the impact of aerobic exercise in people receiving maintenance hemodialysis on various symptoms, including restless legs syndrome, sleep disturbance, anxiety, depression, muscle cramping, and fatigue. Meta-analysis identified a statistically and clinically significant decrease in symptoms of depression measured using the Beck Depression Inventory with exercise compared with controls (mean difference −7.57; 95% confidence interval [CI]: −8.25 to −6.89). In addition, review findings suggested aerobic exercise also improves restless legs syndrome, muscle cramping, and fatigue.^18^ Similarly, a recent Cochrane review and meta-analysis, involving 89 studies and 4291 randomized participants, found that any exercise (aerobic, resistance, or combined aerobic and resistance) probably improves depressive symptoms (10 studies, 441 participants: standardized mean difference [SMD] −0.65, 95% CI: −1.07 to −0.22; I^2^ = 77%) with moderate certainty. The effect of exercise on symptoms of depression was noted to be greater when the exercise intervention was longer than 4 months (6 studies, 311 participants: SMD −0.30, 95% CI: 0.14 to −0.74; I^2^ = 71%). Meta-analysis may improve pain (15 studies, 872 participants: MD 5.28 95% CI: –0.12 to 10.69; I^2^ = 63%). The authors also noted that exercise may improve fatigue with low certainty but data could not be pooled for meta-analysis.^19^

Despite emerging evidence for benefit to several symptoms mentioned previously, the impact of exercise on common hemodialysis-related symptoms (nausea, dyspnea, lack of sleep, and anxiety) is unknown or uncertain and no study has explicitly examined the effects of exercise on overall symptom burden in hemodialysis as a primary outcome, using a validated measurement tool. Only a single study investigated the effect of exercise on dialysis-related symptom burden using a questionnaire with limited previous validation and showed no statistically significant change in symptom burden in individuals participating in a 12-week aerobic and resistance exercise program as compared with non-exercise controls.^20^ Using the Dialysis Symptom Index (DSI), a measurement tool for symptom burden that has been validated in the hemodialysis population, we aim to further characterize the effect of exercise on symptom burden in people receiving facility-based maintenance hemodialysis.

The primary objective of this study is to determine the effect of an exercise rehabilitation program consisting of structured lifestyle education, resistance exercise, and intradialytic cycling on symptom burden at 12 weeks in adult individuals on maintenance hemodialysis. Secondary objectives include examining the effects of the exercise rehabilitation on symptom burden at 26 weeks and 52 weeks, HRQOL and physical activity behavior, and correlating the change in symptom burden with change in HRQOL at all study time points.

## Methods

### Design and Setting

**Figure 1.**
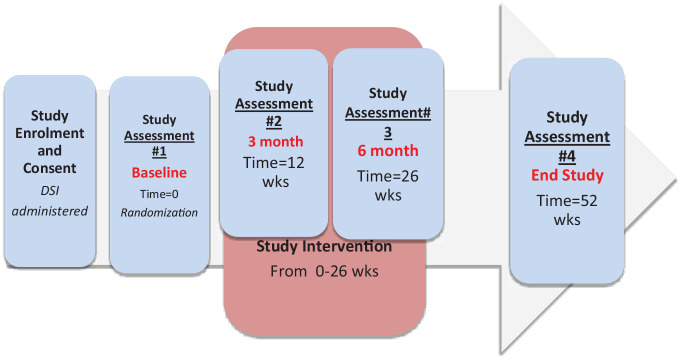
Schematic of study design.

This study is a phase 4, multicenter randomized controlled, 1:1 parallel, open label, blinded prospective end point trial of prevalent adults receiving maintenance hemodialysis at 3 facility-based hemodialysis units (Seven Oaks General Hospital Hemodialysis Units, Sherbrook Centre Hemodialysis Unit, and Health Sciences Centre Central Dialysis Unit) in Winnipeg, Manitoba, Canada (Figure 1).

### Participants

#### Eligibility criteria

Inclusion criteria include adults (aged >18 years) who are >3 months into maintenance hemodialysis, have no expected change in dialysis modality or relocation outside of Winnipeg during the intervention period (26 weeks), are assessed to be able to exercise safely by the hemodialysis unit nephrologist, are able to understand and provide written informed consent, and have at least 1 dialysis-related symptom as indicated by the DSI severity score >0.

Exclusion criteria include those with acute coronary syndrome in the past 3 months, unstable arrhythmia, shortness of breath at rest or with minimal activity (New York Heart Association Class 4), symptomatic hypoglycemia (>2×/week in week prior to enrollment), current participation in the Manitoba Renal Program’s clinical intradialytic cycling program (within past 3 months), and DSI severity score = 0 (ie, no symptoms) when administered at time of consent.

### Recruitment

Voluntary written informed consent to contact is obtained from all participants. Following consent to contact, potential participants are contacted by study staff to arrange an initial meeting. At this meeting, the study is briefly described and the applicable inclusion/exclusion criteria are reviewed with the potential participant to cursorily assess their study eligibility. If eligibility criteria are met after this cursory eligibility review and the individual is interested in proceeding, the study staff proceeds with the informed consent process. Participants are given both verbal and written information detailing the study and provided at least 48 hours to consider the risks and benefits associated with participation. Once informed consent is obtained, the DSI is administered to all participants. If participants score “0” on the DSI (ie, no symptoms), they are deemed ineligible for the study. Once final eligibility is confirmed with the DSI, baseline assessment at the individual’s hemodialysis unit is arranged, at which time all participants receive standardized physical activity counseling (usual care) and undergo baseline testing. This baseline appointment serves as a *“run-in phase*.” Nonattenders are not enrolled. No exercise run-in period was included.

### Randomization

Randomization to the intervention or control arm is performed by a third party using block randomization with randomly permuted block sizes with overall 1:1 allocation. To ensure allocation concealment and to minimize bias, the randomization code is not released until baseline testing is completed. Due to the nature of the intervention in this trial, the study principal investigator (PI), who is a physician working in the hemodialysis units, and study participants cannot be blinded to the study arm. However, outcome assessors and the study statistician are blinded to study group.

## Intervention/Control

As per recommendations, all participants receive standardized exercise counseling at baseline.^21,22^

### Control Arm

Although not prohibited from participating in exercise outside of the study protocol, participants in the control group do not undergo a formal structured exercise intervention and are asked to refrain from intradialytic cycling during the 26-week intervention period. Exercise activity is tracked through weekly self-reported log sheets. The study kinesiologist (to control for personal trainer effect) collects log sheets weekly during the intervention phase and then bimonthly until study end at 1 year.

### Intervention Arm

**Figure 2.**
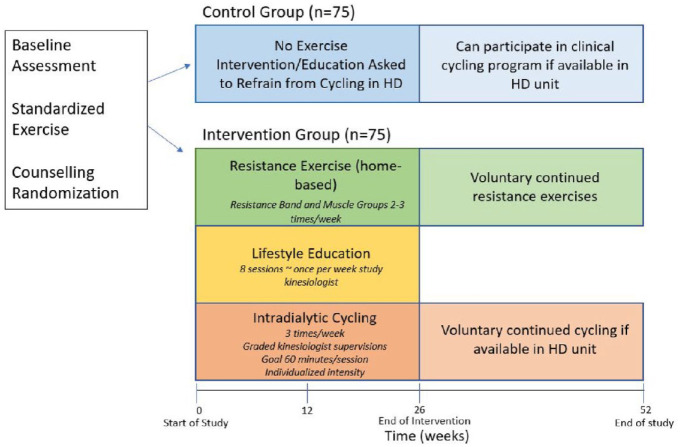
Summary of exercise rehabilitation. HD, hemodialysis.

To standardize intervention exercise intensity, a maximal incremental cycle test (to volitional exhaustion)^23^ is performed on an ergometer prior to hemodialysis on a midweek dialysis day. Participants then participate in a 26-week exercise rehabilitation program as follows (Figure 2):

1. *Standardized one-on-one lifestyle education sessions at hemodialysis provided by the study kinesiologist*: Self-management exercise education using Wellness Institute (Winnipeg, Manitoba) Cardiac Rehabilitation program modules for 1 hour/week during the first 4 weeks of the intervention. Four additional standardized sessions focused on maintenance of lifelong physical activity are provided over the remaining intervention period.
2. *Resistance training, using tubing and body weight, based on established guidelines*^24,25^: Weekly resistance training education is provided by the study kinesiologist in one-on-one format at hemodialysis during the first 4 weeks of intervention. Resistance training bands, instructions, and logbooks are provided for home exercise (goal 2 sessions/week) during the 26-week intervention period.
3. *Cycling during hemodialysis (intensive and maintenance phase)*: Participants will cycle on TherapyCycle (Greely, Colorado) ergometers modified for dialysis beds or chairs,^26^ with an initial target exercise intensity of 50% to 60% of individual maximal workload as determined by the incremental cycling test.^27,28^ Target exercise duration is 60 minutes in the first half of each hemodialysis session, 3 times per week for 26 weeks. Rest periods are provided as needed if participants are unable to complete 60 minutes of continuous exercise.^28,29^ The duration of total exercise time, average workload (Watts), and self-reported exercise intensity as measured by Borg Rating of Perceived Exertion (RPE) are recorded by participants for each exercise session. Target Borg RPE is 13 (on a 20-point scale), which is considered to be moderate intensity.^30^ Ergometer resistance is adjusted over time by the study kinesiologist as triggered by a 1-point change in Borg RPE (Table 1).

**Table 1.** Goal Intensity, Frequency, and Duration of Components of Exercise Intervention.

Type of intervention	Frequency	Time	Duration	Intensity	Location
One-on-one exercise education	1×/wk	1 hour	8 weeks	Not applicable	HD unit
Resistance exercise	2×/wk	8 muscle groups^a^ goal: 12 to 15 reps/muscle	26 weeks	8 repetition max goal RPE = 12-13	Home
Cycling during HD	3×/wk	Goal: 60 minutes	26 weeks	50%-60% max workload (cycling test) goal RPE = 13	HD unit

*Note*. HD, hemodialysis; RPE = rating of perceived exertion.

a Biceps, triceps, chest muscles, hamstrings, quadriceps, hip extensors and hip abductors.

In the first week of the study for the initial 3 intradialytic exercise sessions, the study kinesiologist is present to supervise exercise. Thereafter, for the subsequent dialysis sessions, members of the research team assist with the setup of the cycle ergometers and monitoring during exercise sessions for the participants randomized to exercise. During the intensive phase of the intervention (weeks 2-12), the study kinesiologist checks on participants during intradialytic cycling weekly (at a minimum) to address issues as needed. Subsequently, during the maintenance phase of the intervention (weeks 13-26), the kinesiologist checks in every 2 weeks to help with intensity adjustment, motivation, and troubleshooting. All exercise activity performed outside of hemodialysis, including resistance training, is tracked weekly by self-report using home log sheets during the intervention phase (weeks 1-26) and then bimonthly until study end (weeks 27-52).

### Exercise Following the Intervention Period

Total study duration for each participant is 1 year (52 weeks). If offered as a clinical program in their hemodialysis unit, participants are able to participate in intradialytic cycling *after the intervention phase (26 weeks)* until study end, regardless of study group (Figure 2).

## Study Outcomes

The primary outcome of the study is *change in symptom burden from baseline to 12 weeks as measured by the DSI Severity Score* and assessed as the proportion of individuals who have a decline of 7 points or more in DSI symptom severity score from baseline to 12 weeks. The DSI is a 30-item self-administered questionnaire with low administrative burden developed to measure the presence (yes/no) and severity (“How much did it bother you” by 5-point Likert scale ranging from *not at all* to *very much*) of common symptoms over the previous week in individuals receiving maintenance hemodialysis.^31^ Symptom severity scores can range from 0 to 150. A 7-point change in severity score is likely to be clinically significant because this magnitude of change (1) is consistent with the resolution of 1 “very bothersome” symptom plus a 1-point change in severity of 2 symptoms and likely to be significant to patients (where “very bothersome” symptom equals 5 points), and (2) is approximately 1 SE of the mean based on our pilot data (SD 30, n = 18) and consistent with distribution-based methods. The DSI has been shown to be reliable in predicting HRQOL in multiple studies in North American and European hemodialysis population.^7,31-33^ To our knowledge, the DSI has not been used in any other interventional exercise studies. One small cross-sectional observational study investigating the relationship between physical activity level and symptom burden in 48 prevalent individuals receiving hemodialysis demonstrated that each 1000 step count increase in physical activity was associated with statistically significant decreases in the severity of fatigue and insomnia measured by the DSI.^34^

See Table 2 for secondary and exploratory outcomes.

**Table 2.** Study Secondary Outcomes.

Secondary outcomes	
1	Change in symptom burden from baseline to 12 weeks as measured by modified DSI Severity Score, a cluster of symptoms that are most plausible to benefit from exercise and include (1) muscle cramps, (2) restless legs, (3) feeling tired or lack of energy, (4) difficulty concentrating, (5) worrying, (6) feeling nervous, (7) trouble falling and/or staying asleep, (8) feeling irritable, (9) feeling sad, and (10) feeling anxious
2	Change in generic HRQOL from baseline to 12, 26, and 52 weeks assessed using the EQ-5D-5L and EQ Visual Analogue Score (VAS)15,^35-37^
3	Change in time required to recover from hemodialysis (in minutes) from baseline to 12, 26, and 52 weeks using the question “Approximately how much time does it take to recover from a dialysis session?”^38,39^
4	Change in symptom burden from baseline to 26 and 52 weeks as measured by the Dialysis Symptom Index (DSI) Severity Score^7,31-33^
5	Change in physical activity behavior patterns from baseline to 12, 26, and 52 weeks measured objectively using triaxial accelerometry and by self-report using the Godin-Shephard Leisure-Time Exercise Questionnaire^40,41^
6	Adherence to study intervention measured using a. Proportion of resistance and intradialytic cycling exercise sessions logged compared with total number of possible exercise sessions. b. Proportion of total minutes of intradialytic cycling achieved based on logbook records compared with goal intradialytic cycling minutes.
7	Change in Exercise Capacity from baseline to 12, 26, and 52 weeks as measured in meters by the Incremental Shuttle Walk Test (ISWT)^42-45^
8	Change in frailty status from baseline to 12, 26, and 52 weeks assessed using the Modified Fried Criteria defined as the presence of ≥3 criteria of the following criteria: weight loss, exhaustion, slowness, low physical activity, and weakness.^46,47^
9	Change in self-efficacy for exercise from baseline to 12, 26, and 52 weeks assessed using the Self-Efficacy for Exercise Survey^48-51^
Exploratory outcomes	
1	Hospitalization rate defined as the number of hospitalization days admitted per patient year
2	All-cause mortality defined as the proportion of patients who die over 1 year and cause of death obtained from dialysis unit records

*Note*. EQ = EuroQol; HRQOL, health-related quality of life; VAS = visual analogue score; ISWT = incremental shuttle walk test.

## Data Collection Procedures and Timing

*Demographic Data* are collected by a blinded assessor at baseline using patient interview and hemodialysis chart review, and includes age, sex, race, cause of kidney failure, dialysis vintage (time on hemodialysis) comorbidities, use of walking aid (type), and smoking history.

*Clinical Data* are collected by a blinded assessor at baseline, 12-, 26-, and 52-week assessments via hemodialysis chart review and includes medications (weekly erythropoietin stimulating agent dose, blood pressure and cardiac medications and dosage), dialysis adequacy (mean Kt/V for 3 hemodialysis sessions preceding study assessment), pre-hemodialysis and post-hemodialysis sitting systolic and diastolic blood pressure and heart rate, dialysis prescription (dialysis duration, frequency of hemodialysis sessions, dialysate potassium, sodium and calcium, and dry weight), and mean fluid gain (total volume [L]) of fluid gained between interdialytic sessions in the 3 hemodialysis sessions before assessment.

*Laboratory Data*, including hemoglobin, albumin, potassium, calcium, phosphate, and parathyroid hormone, taken at the closest time point to each study assessment visit is collected through hemodialysis chart review of monthly blood work taken routinely as part of standard clinical care pre-dialysis on a midweek dialysis day.

*Outcome Assessments* occur at baseline, 12, 26, and 52 weeks (within +/–10 days of this time point) with are conducted by a blinded assessor and are performed pre-dialysis on a midweek dialysis day to minimize the effects of fluid overload and dialysis fatigue but maximize convenience and adherence to the assessment schedule (Table 3). The baseline assessment, which occurs before randomization when the study group is still unknown, is performed by the study kinesiologist to allow for baseline exercise counseling. Subsequent appointments are conducted by a blinded assessor as the study kinesiologist can no longer be blinded. All demographic, clinical, laboratory, and outcome assessment data are entered into a secure REDCap database.

**Table 3.** Data Collection Plan.

Scheduled study procedure	Screening/enrollment (visit 0)	Baseline (visit 1)	12 weeks (visit 2)	26 weeks (visit 3)	Final visit 52 weeks (visit 4)
Informed consent	x				
Final study eligibility	x	x			
Demographics		x			
Medical history		x			
Physical/clinical assessment		x	x	x	x
Concomitant medications		x	x	x	x
Safety monitoring adverse events		x	x	x	x
Protocol compliance		x	x	x	x
Dialysis Symptom Index	x	x	x	x	x
EQ-5D-5L/EQVAS		x	x	x	x
Time to recover from HD		x	x	x	x
Accelerometry		x	x	x	x
Godin-Shephard Leisure Time Exercise Questionnaire		x	x	x	x
Incremental Shuttle Walk Test		x	x	x	x
Frail status		x	x	x	x
Self-efficacy for exercise study		x	x	x	x
Hospitalization history			x	x	x
Death			x	x	x
Collection of exercise logs		Weekly		Every 2 weeks	
Incremental Cycle Test	(X)^a^				

*Note*. EQ = EuroQol; EQVAS, EuroQol Visual Analogue Scale.

a In intervention group, only between enrollment visit and start of intervention.

### Sample Size and Power

Based on the limited literature published in this area, we expect an absolute difference of 25% between the intervention and control groups in the proportion of individuals in whom the DSI severity score decreases by 7 points or more over time. We predict DSI score will decrease in 50% of the intervention group and 25% of the control group at 12 weeks (baseline prevalence of symptoms assumed to be 50%). A sample size of 58 patients in each arm will allow us to detect this difference with 80% power (α = 0.05). We conservatively anticipate 30% dropout.^29^ Therefore, a sample size of 150 participants (75 participants per study arm) was originally targeted. This sample size will also allow us to detect a 7-point difference in mean change (assuming 16 SD) in DSI severity score between groups with 80% power and α = 0.05.

To account for difficulties with recruitment, implementation of exercise intervention, outcome assessments, and increased study dropouts due to COVID-19 pandemic restrictions, study recruitment was increased to 193 individuals.

### Ethical Issues

This study is conducted in compliance with International Council for Harmonization of Technical Requirements and the Declaration of Helsinki.^52^ The study protocol and informed consent forms have been approved by the University of Manitoba Biomedical Research Ethics Board no. HS18243 (B2014:088). The trial is registered at the US National Institutes of Health (ClinicalTrials.gov) no. NCT02259413.

## Statistical Analyses

A statistician will perform a blinded analysis in an intention-to-treat manner on a case available basis. We will compare baseline clinical and demographic variables and outcome data between study groups using independent 2-tailed *t* test or Mann-Whitney U test, depending on data distribution, for continuous variables and χ^2^ test for categorical variables, as appropriate. Similarly, individuals who complete the study protocol will be compared with those who do not.

Change in DSI severity score from baseline to 12 weeks will be compared between groups by independent 2-tailed *t* test or Mann-Whitney U test depending on distribution and using generalized linear mixed models with study timepoint as a random effect and adjusted for baseline DSI score. In a subgroup of individuals whose baseline DSI score is >7, the proportion of individuals whose DSI severity score has improved by 7 or more points from baseline to 12 weeks will be compared between groups at each study time point using χ^2^ test.

Multiple logistic regression will be performed to determine predictors of a >7-point change in DSI severity score over time and adjusted for time-varying confounders using a marginal structural model. Interaction terms for sex and baseline DSI score will also be inputted into the model. Although the study group will be the primary predictor in this model, other key predictors of symptom burden, including baseline DSI score, diabetes status, ischemic heart disease (IHD), age, sex, race, smoking status, serum albumin level, hemoglobin, baseline physical function, physical activity level, and interaction terms for gender/intervention and baseline DSI score/intervention, will also be tested in a stepwise manner.

*Planned Subgroup Analyses* include analysis of primary outcome by gender and quartiles of baseline physical activity level as measured by accelerometry at baseline. Nonadherence to the exercise intervention may modify the effect of the intervention and is an important potential confounder. Therefore, a prespecified analysis of the primary outcome will be performed “on protocol,” defined by adherence to intervention protocol as defined by completion of 70% or greater of exercise sessions during the intervention period.

### Secondary Outcomes Analyses

We will repeat the primary outcome analyses comparing change in DSI and modified DSI between study groups from baseline to 26 weeks and 52 weeks and between each time period. Change in EQVAS over time will be compared between study groups using Student *t* test. The difference in proportion of individuals who develop new deficits (score of 2 or greater) in each domain of the EQ-5D-5L over time will also be compared. The degree to which change in symptom burden correlates with change in EQVAS will be assessed using Spearman’s and Pearson’s correlation coefficients as indicated based on distribution of the data. Change in all other secondary outcomes over time will be compared between groups using the appropriate parametric and nonparametric statistical tests, depending on data type and distribution. Bonferroni adjustments will be made for secondary outcomes to adjust for multiple outcomes and study time points. Hospitalization rate will be analyzed using Poisson regression. For all analyses, 2-sided *P* < .01 will be statistically significant. Statistical analysis will be performed using SAS 9.3 (Carey, North Carolina).

### The COVID-19 Pandemic–Related Protocol Adaptations

The COVID-19 pandemic and related restrictions significantly affected the implementation, conduct, and completion of this trial. As per University of Manitoba policies, recruitment for the study was halted between March 2020 and June 2021. This delayed the completion of study recruitment. Due to the requirement that only essential health care personnel enter hemodialysis units, study kinesiologists and staff were unable to interact and perform study assessments in person with study participants between March 2020 and June 2021. As a result, some cycling sessions during this period were missed and accelerometry and physical function outcomes were not collected. Fortunately, we were able to continue collecting survey data including our primary outcome by completing questionnaires over the phone with participants. An additional consequence of the COVID-19 pandemic–related hiatus to in-person study visits was the higher than anticipated participant dropout during this period. In response, we reopened recruitment in February 2022 and consented an additional 13 (total of 193) participants to ensure adequate power for the study. See Table 4 for all study modifications that were required related to COVID-19 pandemic restrictions.

**Table 4.** COVID-19 Pandemic–Related Changes in Study Operating Procedures.

Study activity	Pre-usual procedure–COVID 19	Modification for COVID-19 pandemic restrictions post-COVID 19
Recruitment	Approach patients in hemodialysis unit	On hold from March 2020 to June 2021
Activity logs	Study kinesiologist visited participant in hemodialysis unit and collected activity information	Study kinesiologist collected information through phone or email
Outcome assessments	Assessment appointments scheduled with participant in person	In-person assessments on hold from March 2020 to June 2021. Only components of the assessment that were possible by phone were completed (eg, questionnaires) during this time
Accelerometers	Accelerometers were provided to the study participant to wear 1 week prior to study assessment date	Accelerometers were not provided between March 2020 and June 2021 to avoid potential transmission of COVID-19
Participant reimbursement	Participants were given $20 at each in-person study assessment	Participants were mailed a $25 VISA/Mastercard gift card
Data collection	Data collection was completed in real time in the hemodialysis units	Data collection was on hold until research staff were able to return to work on dialysis units
Personal protective equipment (PPE)	Research staff did not wear PPE	Research staff were required to wear proper PPE including face mask, protective eye wear, and gown as well as maintain social distance (6 ft)

### Data Safety Monitoring Board

A Data Safety Monitoring Board (DSMB) reviews safety data yearly and makes recommendations as to whether the study should continue as is or be modified to protect participants’ safety, or be terminated. The DSMB works independently from the trial and did not participate in development of the statistical plan.

## Discussion

Individuals receiving hemodialysis have high symptom burden, low functional status, and poor HRQOL, for which effective treatments known to impact clinical outcomes are extremely limited. The identification of effective treatments for dialysis-related symptoms has been identified as a research priority by hemodialysis patients, their caregivers, and health care providers in Canada. In other chronic disease programs, exercise rehabilitation programs, incorporating resistance and aerobic exercise and lifestyle education, have demonstrated benefits to outcomes closely associated with symptom burden, such as physical function and HRQOL.^34,52^

One previous RCT has examined the effect of exercise on overall symptom burden in individuals receiving hemodialysis, using a questionnaire with limited previous validation. In this study, 96 individuals were randomized to an exercise intervention (n = 53) and a control group (n = 43) consisting of 12 weeks of pre-dialysis resistance exercise and intradialytic cycling or usual care controls. No statistically significant difference in change in symptom burden was seen in the exercise group as compared with non-exercise controls.^20^

With randomization of 150 individuals with symptoms receiving maintenance hemodialysis and using a validated tool to measure symptom burden, this study will provide important evidence regarding the benefits of exercise rehabilitation on symptom burden in people receiving hemodialysis. This pragmatic study prioritizes real-world applicability by employing broad eligibility criteria for a representative sample, utilizing a flexible, individualized intervention seamlessly integrated into hemodialysis treatments without added participant time, and assessing clinically relevant and patient-important outcomes. As the largest study to examine the effect of exercise on symptom burden as a primary outcome and with its pragmatic approach, individualized exercise intervention, and plan to correlate change in symptom burden with change in HRQOL, the findings of this study will be applicable in clinical practice and relevance, and generalizable to the broader maintenance hemodialysis population.

We have implemented the following steps to mitigate potential challenges to study implementation and address study limitations. To maximize generalizability, we selected clinically relevant inclusion criteria based on input from clinicians and clinical trialists. We also included a “run-in” phase to minimize study dropout and estimated a conservative 30% dropout rate.^29^ The same third-party assessor, blinded to study group, performs all outcome assessments following the baseline assessment to minimize bias. Although blinding may be incomplete due to the nature of the intervention (research staff may be on the unit and see intervention participations), we ask participants to refrain from communicating which study group they were assigned to the assessor. The study statistician will be blinded to study group during analysis. In addition, exercise in the control group may dilute differences observed between study groups. With known benefits of exercise in this population, it was unethical to ask individuals in the control group to remain sedentary for the duration of the study. Physical activity behavior measured objectively using accelerometry will be incorporated into regression modeling to assess the impact of this potential modifier on study outcomes and facilitate calculation of activity dose-response.

Finally, the primary outcome measure, the DSI severity score, is subject to measurement error and noise related to the 30-symptom inventory it measures and its limited ability to define outcome severity with a Likert scale. However, there is no perfect solution. Other tools to measure symptom burden in hemodialysis are no better. We anticipate that benefit from exercise will occur in a subset of symptoms on the DSI. We considered creating a new composite outcome of those 10 symptoms (muscle cramps, restless legs, feeling tired or lack of energy, difficulty concentrating, worrying, feeling nervous, trouble falling and/or staying asleep, feeling irritable, feeling sad, and feeling anxious) but had concerns regarding the validity of the use of a novel measure with no prior validation as our primary outcome. We selected the DSI due to its extensive previous use and demonstrated association with HRQOL, an outcome that has been shown to improve with exercise programming. To address the above concerns, we have included the modified DSI severity score as a secondary outcome, which includes the 10 symptoms listed above and will lead to less participant burnout than if individual measures are used for each symptom.

In conclusion, the application of an exercise rehabilitation program to improve symptom burden in individuals on hemodialysis is novel, may ameliorate common symptoms observed in individuals on hemodialysis, and result in improved quality of life and reduced disability and morbidity over the long term. Importantly, this pragmatic study, with a standardized exercise intervention, is patient-informed, adaptable to baseline physical function, and addresses important gaps in both clinical care of people receiving hemodialysis and our current knowledge.

## Supplemental Material

sj-docx-1-cjk-10.1177_20543581241234724 – Supplemental material for Randomized Controlled Trial of the Effect of an Exercise Rehabilitation Program on Symptom Burden in Maintenance Hemodialysis: A Clinical Research Protocol Supplemental material, sj-docx-1-cjk-10.1177_20543581241234724 for Randomized Controlled Trial of the Effect of an Exercise Rehabilitation Program on Symptom Burden in Maintenance Hemodialysis: A Clinical Research Protocol by Emilie Ford, Krista Stewart, Eric Garcia, Monica Sharma, Reid Whitlock, Ruth Getachew, Krista Rossum, Todd A. Duhamel, Mauro Verrelli, James Zacharias, Paul Komenda, Navdeep Tangri, Claudio Rigatto, Jennifer M. MacRae and Clara Bohm in Canadian Journal of Kidney Health and Disease

sj-docx-2-cjk-10.1177_20543581241234724 – Supplemental material for Randomized Controlled Trial of the Effect of an Exercise Rehabilitation Program on Symptom Burden in Maintenance Hemodialysis: A Clinical Research Protocol Supplemental material, sj-docx-2-cjk-10.1177_20543581241234724 for Randomized Controlled Trial of the Effect of an Exercise Rehabilitation Program on Symptom Burden in Maintenance Hemodialysis: A Clinical Research Protocol by Emilie Ford, Krista Stewart, Eric Garcia, Monica Sharma, Reid Whitlock, Ruth Getachew, Krista Rossum, Todd A. Duhamel, Mauro Verrelli, James Zacharias, Paul Komenda, Navdeep Tangri, Claudio Rigatto, Jennifer M. MacRae and Clara Bohm in Canadian Journal of Kidney Health and Disease

sj-docx-3-cjk-10.1177_20543581241234724 – Supplemental material for Randomized Controlled Trial of the Effect of an Exercise Rehabilitation Program on Symptom Burden in Maintenance Hemodialysis: A Clinical Research Protocol Supplemental material, sj-docx-3-cjk-10.1177_20543581241234724 for Randomized Controlled Trial of the Effect of an Exercise Rehabilitation Program on Symptom Burden in Maintenance Hemodialysis: A Clinical Research Protocol by Emilie Ford, Krista Stewart, Eric Garcia, Monica Sharma, Reid Whitlock, Ruth Getachew, Krista Rossum, Todd A. Duhamel, Mauro Verrelli, James Zacharias, Paul Komenda, Navdeep Tangri, Claudio Rigatto, Jennifer M. MacRae and Clara Bohm in Canadian Journal of Kidney Health and Disease
