# Supplementary material for: Randomized Controlled Trial of the Effect of an Exercise Rehabilitation Program on Symptom Burden in Maintenance Hemodialysis: A Clinical Research Protocol
Source: Can J Kidney Health Dis. 2024 Apr 3;11:20543581241234724. doi: 10.1177/20543581241234724 (PMC10993676; doi:10.1177/20543581241234724)
Supplement: sj-docx-2-cjk-10.1177_20543581241234724 – Supplemental material for Randomized Controlled Trial of the Effect of an Exercise Rehabilitation Program on Symptom Burden in Maintenance Hemodialysis: A Clinical Research Protocol [file sj-docx-2-cjk-10.1177_20543581241234724.docx]

**Supplemental Table 2.** CONSERVE-SPIRIT checklist

| CONSERVE-SPIRIT Extension: [DATE] | | | | | |
| --- | --- | --- | --- | --- | --- |
| Item | Item Title | Description | | | Page No. |
| I. | Extenuating Circumstances | Describe the circumstances and how they constitute extenuating circumstances. | | | 13 |
| II. | Important Modifications | 1. Describe how the modifications are important modifications. | | | 13 |
|  |  | 1. Describe the impacts and mitigating strategies, including their rationale and implications for the trial. | | | 13, 25 |
|  |  | 1. Provide a modification timeline. | | | 13, 25 |
| III. | Responsible Parties | State who planned, reviewed and approved the modifications. | | | 13 |
| IV. | Interim data | If modifications were informed by trial data, describe how the interim data were used, including whether they were examined by study group, and whether the individuals reviewing the data were blinded to the treatment allocation. | | | N/A |
| SPIRIT Item and Number | | For each row, if important modifications occurred, check one or both of “impact” and/or “mitigating strategy” and describe the changes in the protocol. Check “no change” for items that are unaffected in the extenuating circumstance. | | | Page No. |
|  |  | No Change | Impact* | Mitigating Strategy** |  |
| 1 | Title | *✓* |  |  |  |
| 2 | Trial registration | *✓* |  |  |  |
| 3 | Protocol version | *✓* |  |  |  |
| 4 | Funding | *✓* |  |  |  |
| 5 | Roles and responsibilities |  | *✓* | Updated roles to reflect COVID-19 restrictions | 13, 25 |
| 6 | Background and rationale | *✓* |  |  |  |
| 7 | Objectives | *✓* |  |  |  |
| 8 | Trial design | *✓* |  |  |  |
| 9 | Study setting |  | *✓* | Unable to enter units so completed questionnaires over phone | 13, 25 |
| 10 | Eligibility criteria | *✓* |  |  |  |
| 11 | Interventions |  | *✓* | Cycling sessions missed because research staff not allowed in hemodialysis units | 13, 25 |
| 12 | Outcomes |  | *✓* | Outcomes requiring in person assessments ON HOLD during COVID 19 | 13, 25 |
| 13 | Participant timeline | *✓* |  |  |  |
| 14 | Sample size | *✓* |  |  |  |
| 15 | Recruitment |  | *✓* | ON HOLD during COVID 19  Recruitment delayed | 13, 25 |
| 16 | Allocation | *✓* |  |  |  |
| 17 | Blinding (masking) | *✓* |  |  |  |
| 18 | Data collection methods |  | *✓* | ON HOLD during COVID 19 | 13, 25 |
| 19 | Data management | *✓* |  |  |  |
| 20 | Statistical methods | *✓* |  |  |  |
| 21 | Data monitoring | *✓* |  |  |  |
| 22 | Harms | *✓* |  |  |  |
| 23 | Auditing | *✓* |  | Delayed until research staff could enter units to review hemodialysis charts |  |
| 24 | Research ethics approval | *✓* |  |  |  |
| 25 | Protocol amendments |  | *✓* | Submitted COVID-19 amendment | 13, 25 |
| 26 | Consent or assent |  | *✓* | ON HOLD during COVID 19 | 13, 25 |
| 27 | Confidentiality | *✓* |  |  |  |
| 28 | Declaration of interests | *✓* |  |  |  |
| 29 | Access to data | *✓* |  |  |  |
| 30 | Ancillary and post-trial care | *✓* |  |  |  |
| 31 | Dissemination policy | *✓* |  |  |  |
| 32 | Informed consent materials |  | *✓* | ON HOLD during COVID 19 | 13, 25 |
| 33 | Biological specimens | *✓* |  |  |  |
| *Aspects of the trial that are directly affected or changed by the extenuating circumstance and are not under the control of investigators, sponsor or funder.  **Aspects of the trial that are modified by the study investigators, sponsor or funder to respond to the extenuating circumstance or manage the direct impacts on the trial. | | | | | |
