# Supplementary material for: Randomized Controlled Trial of the Effect of an Exercise Rehabilitation Program on Symptom Burden in Maintenance Hemodialysis: A Clinical Research Protocol
Source: Can J Kidney Health Dis. 2024 Apr 3;11:20543581241234724. doi: 10.1177/20543581241234724 (PMC10993676; doi:10.1177/20543581241234724)
Supplement: sj-docx-3-cjk-10.1177_20543581241234724 – Supplemental material for Randomized Controlled Trial of the Effect of an Exercise Rehabilitation Program on Symptom Burden in Maintenance Hemodialysis: A Clinical Research Protocol [file sj-docx-3-cjk-10.1177_20543581241234724.docx]

**Supplemental Table 3.** CERT Checklist – Consensus on Exercise Reporting Template

| Section/Topic | Item # | Checklist item | Location ** | |
| --- | --- | --- | --- | --- |
|  |  |  | Primary paper (page, table, appendix) | † Other (paper or protocol, website(URL) |
| WHAT: materials | 1 | Detailed description of the type of exercise equipment (e.g. weights, exercise equipment such as machines, treadmill, bicycle ergometer etc) | 2, 8-9 |  |
| WHO: provider | 2 | Detailed description of the qualifications, teaching/supervising expertise, and/or training undertaken by the exercise instructor | 8-9 |  |
| HOW: delivery | 3 | Describe whether exercises are performed individually or in a group | 8-9 |  |
|  | 4 | Describe whether exercises are supervised or unsupervised and how they are delivered | 8-9 |  |
|  | 5 | Detailed description of how adherence to exercise is measured and reported | 8-9 |  |
|  | 6 | Detailed description of motivation strategies | 8-9 |  |
|  | 7a | Detailed description of the decision rule(s) for determining exercise progression | 8-9 |  |
|  | 7b | Detailed description of how the exercise program was progressed | 8-9 |  |
|  | 8 | Detailed description of each exercise to enable replication (e.g. photographs, illustrations, video etc) | Not provided |  |
|  | 9 | Detailed description of any home program component (e.g. other exercises, stretching etc) | 8 |  |
|  | 10 | Describe whether there are any non-exercise components (e.g. education, cognitive behavioural therapy, massage etc) | 8 |  |
|  | 11 | Describe the type and number of adverse events that occurred during exercise | N/A – Protocol only |  |
| WHERE: location | 12 | Describe the setting in which the exercises are performed | 8-9 |  |
| WHEN, HOW  MUCH: dosage | 13 | Detailed description of the exercise intervention including, but not limited to, number of exercise repetitions/sets/sessions, session duration, intervention/program duration etc | 8-9; Table 1 |  |
| TAILORING:  what, how | 14a | Describe whether the exercises are generic (one size fits all) or tailored whether tailored to the individual | 8-9 |  |
|  | 14b | Detailed description of how exercises are tailored to the individual | 8-9 |  |
|  | 15 | Describe the decision rule for determining the starting level at which people commence an exercise program (such as beginner, intermediate, advanced etc) | 8-9 |  |
| HOW WELL: planned, actual | 16a | Describe how adherence or fidelity to the exercise intervention is assessed/measured | 8-9 |  |
|  | 16b | Describe the extent to which the intervention was delivered as planned | N/A Protocol Only |  |
